# Establishing a National Emergency Department Surveillance: an innovative study from Pakistan

**DOI:** 10.1186/1471-227X-15-S2-I1

**Published:** 2015-12-11

**Authors:** Prasanthi Puvanachandra, Junaid A Razzak, Adnan A Hyder

**Affiliations:** 1 Johns Hopkins International Injury Research Unit, Department of International Health, Johns Hopkins Bloomberg School of Public Health, Baltimore, Maryland, USA; 2 Department of Emergency Medicine, Johns Hopkins School of Medicine, Baltimore, Maryland, USA; 3 The author was affiliated with the Department of Emergency Medicine, Aga Khan University, Karachi, Pakistan at the time when study was conducted

## 

Low and Middle Income Countries (LMIC) are facing an epidemiological transition from communicable to non-communicable diseases challenging public health agencies which traditionally have concentrated on maternal or infectious (HIV or malaria) diseases [1,2]. At the same time, while prevention resides at the core of any health system, provision of emergency care for time-sensitive illnesses remains a necessity and yet is not a priority for many health systems in LMIC [3]. Emergency care makes a substantial contribution to reducing mortality and morbidity in a population, and is beginning to gain some global attention amongst policymakers in LMIC [4-6].

The traditional view that emergency care is peripheral and costly is contestable since empirical data demonstrates that simple, effective strategies can be utilized [7]. Myths that emergency care is only focused on ambulance provision and transport, neglect the profound role that it can have within both the community and healthcare facilities also needs to be debunked through the provision of scientific evidence [6].

Emergency departments (ED) encounter a wide array of diseases from communicable infections to mass-scale trauma; with such a large spectrum of disease, it is immensely challenging to accurately define the burden posed on an emergency department. There is a dearth of such information, particularly in LMIC, surrounding the epidemiology of emergency conditions which in turn limits the degree to which appropriate policy and health system development can be informed. This is particularly true in LMIC like Pakistan where basic disease burden information within EDs is not available. Given that EDs are such a critical component of any healthcare system, it is imperative to ensure that this evolution, particularly within LMIC is based on a sound understanding of the epidemiology of conditions being addressed by such systems.

This special issue of this journal focuses on the development and pilot testing of the Pakistan National Emergency Department Surveillance System (Pak-NEDS) within seven major tertiary healthcare centers across Pakistan. A full description of how this surveillance system was piloted is presented in "The Pakistan National Emergency Department Surveillance Study (Pak-NEDS): Introducing a pilot surveillance". The remaining papers present key findings from the first round of data collection. The feasibility and benefits of piloting such a surveillance system to accurately quantify the burden of diseases requiring emergency care and to guide prevention efforts is demonstrated throughout these papers.

Data presented in the poisoning study revealed that over half of all poisoning cases presenting to the ED were intentional and 44% were due to ingestions or inhalation of poisonous chemicals. These findings clearly indicate the necessity for the provision of improved mental health services and regulatory control over hazardous chemicals in Pakistan.

Five percent of all injury patients (N = 68,390) registered through Pak-NEDS incurred a fall-related injury with 70% of patients being within the productive age-group of 15-44 years. Studies such as "Pattern of fall injuries in Pakistan: The Pakistan National Emergency Department Surveillance (Pak-NEDS) study" help address the paucity of information surrounding fall-related injuries and demonstrate the need for future research to be focused on safety measures both at home and within the workplace.

Whilst drowning remains a heavy burden on health systems in LMIC, limited data very often leads to ineffective prevention strategies. The study entitled "Pattern of Presenting Complaints Recorded as Near-Drowning Events in Emergency Departments: A National Surveillance Study from Pakistan" presented in this special issue reveals that even with the existence of surveillance systems such as Pak-NEDS there is still a major underreporting of drowning and near-drowning events. Greater emphasis needs to be placed on data source selection, site locations, ED care standards and multisector collaboration in Pakistan.

Estimating the true burden of burn injuries, particularly with limited or no specialized tertiary burn centers is extremely difficult without adequate surveillance. Scalds and flame burns were the commonest cause of burn injury (70% and 18% respectively) of those discharged from the ED as highlighted in "Burn injury characteristics: Findings from Pakistan National Emergency Department Surveillance Study". Such data further serves to reinforce knowledge on prevention strategies surrounding the use of kerosene stoves and wood-based cooking fuel.

EDs offer an excellent opportunity to collect epidemiological data on intentional injuries among children [8]. The paper in this special issue focusing on child violence reports that of the 30.937 children who were registered through Pak-NEDS, 8.2% were intentionally injured. The majority of these injuries were as a result of assault (65.8%) followed by self-inflicted injuries (34.2%). This paper highlights the role that active, sustainable surveillance systems such as Pak-NEDS can have in raising the awareness of violence against children and directing prevention programs towards this public health priority.

The burden on Pakistan's healthcare systems due to bomb blast injuries is explored in "Bomb blast injuries: an exploration of patient characteristics and outcome using Pakistan National Emergency Departments Surveillance (Pak-NEDS) data". The majority of the patients were young males predominantly in Peshawar and Karachi. Utilization of ambulance services was low. Such findings highlight the need for strengthened pre-hospital services and challenges faced by EDs in responding to such events.

The use of Pak-NEDS data to describe the extent and characteristics of patients who are dead on arrival (DOA) from the perspective of pre-hospital and ED care is presented in this special issue. Such data is instrumental in promoting simple, cost-effective pre-hospital care systems. A second paper on pre-hospital care entitled "Ambulance use in Pakistan: An analysis of surveillance data from emergency departments in Pakistan" focuses on examining the use of ambulance services in Pakistan. Of the patients where mode of transport information was available, only four percent were brought to the ED by ambulance highlighting the underutilization of such services.

One of the core requirements of any emergency system is to be able to provide effective triage of patients as they enter the ED. Only one of the seven hospitals involved with the Pak-NEDS study had a formal triage system in place, with the others relying on a first-come first-serve basis. In "How vital are the vital signs?" the use of vital signs to help guide triage decisions, resource allocations and ultimately improve patient mortality is explored.

The potential development of specific pediatric emergency care departments and guidelines for the commonest causes of pediatric illness presenting to EDs is discussed in "The Pediatric Disease Spectrum in Emergency Departments across Pakistan: Data from a Pilot Surveillance System". This paper reports that pediatric cases represented a tenth of all general ED patients registered through Pak-NEDS with injury-related visits accounting for over half of this group highlighting the need for reinforcement of pediatric trauma services and standardized protocols for violence against children.

The paper focusing on traumatic brain injuries (TBI) assesses emergency care access and out-of-pocket treatment costs associated with TBI in Pakistan. Findings showed that 49% of victims were as a result of road traffic injuries, followed by falls (22%). The overwhelming majority presented to the public hospitals (98%) however access to attending level physicians within the ED was as low as 1% which falls outside international guidelines for care of severe TBI patients. The high costs of TBI imaging and its subsequent clinical implications in LMIC settings is also discussed.

With chest pain being the second most common cause of ED visits, the ability to accurately interpret clinical signs and diagnose underlying conditions is crucial in preventing adverse outcomes. "Burden of chest pain in a low-and middle-income country: analysis of emergency department surveillance data" provides a much needed analysis of the burden of chest pain in a LMIC setting. The limited available data highlights the need for the gap between international guidelines and current practice in Pakistan to be closed.

As healthcare and technology continue to advance, the spectrum of disease being treated within EDs continues to widen. It is therefore imperative that in this context of increasing demand on emergency services, the true burden of disease being treated within EDs needs to be accurately defined. An ED-based surveillance system in a low-income context like Pakistan, allows the generation of such data. The papers in this special issue clearly demonstrate the feasibility of capturing the burden of disease within EDs within limited resource settings. A surveillance system can be set up and implemented in multiple EDs with a central data and reporting center. The challenges can be confronted and this special issue should provide ample lessons for future endeavors.

## Competing interests

The authors declare that they have no competing interests.
